# Siglec-targeted liposomes to identify sialoglycans present on fungal pathogens

**DOI:** 10.1128/aac.01720-24

**Published:** 2025-03-14

**Authors:** Suresh Ambati, Quanita J. Choudhury, Jesse Ann Peter, Kelley W. Moremen, Digantkumar Gopaldas Chapla, Zachary A. Lewis, Xiaorong Lin, Richard B. Meagher

**Affiliations:** 1 Department of Genetics, University of Georgia 189619 , Athens, Georgia, USA; 2 Department of Microbiology, University of Georgia 189270 , Athens, Georgia, USA; 3 Department of Biochemistry and Molecular Biology, University of Georgia 174518 , Athens, Georgia, USA; 4 Complex Carbohydrate Research Center, University of Georgia 123423 , Athens, Georgia, USA; University Children's Hospital Münster, Münster, Germany

**Keywords:** sialic acid, Neu5Ac, sialo-glycoprotein, fungi, pathogens, antifungal agents, liposomes, amphotericin B, targeted, Siglec

## Abstract

The sialic acid Ig-like lectins Siglec-3 and Siglec-15 are pathogen receptors that bind sialic acid-modified glycoproteins, best characterized in metastatic cancers. Because fungi produce sialoglycans and sialo-glycoproteins, we wondered if Siglecs had the potential for targeted delivery of antifungal drugs. We purified the extracellular V-region Ig-like C2 ligand-binding domains and stalk regions of SIG3 and SIG15. We floated the two polypeptides on the surface of liposomes loaded with amphotericin B (AmB) and labeled with rhodamine B to prepare SIG3-Ls and SIG15-Ls. Using these two reagents, we explored the sialoglycans of two evolutionarily distant and deadly human fungal pathogens, the Mucormycete *Rhizopus delemar* and the Ascomycete *Aspergillus fumigatus*. We found that SIG3-Ls and SIG15-Ls localized in a continuous layer over the cell wall surface of germ tubes and hyphae of both fungal species and to the conidia of *A. fumigatus*. Binding was Neu5Ac-specific and appeared confined to N-linked sialoglycans on fungal proteins. SIG3 and SIG15 proteins bound to diverse sialo-glycoproteins extracted from the hyphae of both species. SIG3-Ls and SIG15-Ls delivering sub-micromolar concentrations of AmB were moderately more effective at inhibiting and/or killing both species relative to control liposomes. We discuss the roles that sialo-glycoproteins may play in fungal pathogens.

## INTRODUCTION

Invasive aspergillosis and mucormycosis are two of the deadliest fungal diseases, with treatment mortality rates approaching 70% (1). Globally, they account for more than 2,000,000 and 80,000 deaths each year, respectively. The number of reported cases of aspergillosis and mucormycosis has increased by several fold in the last five decades, paralleling the increasing numbers of individuals with diabetes and those taking immunosuppressants (2–4). *Aspergillus fumigatus* is responsible for the majority of cases of aspergillosis. *Rhizopus delemar* (frequently known as *R. oryzae* and *R. arrhizus*) is responsible for at least half of all diagnosed cases of mucormycosis (5–7). Both are opportunistic pathogens that live in soil on rotting vegetation, which likely accounts for the high rate of infection among agricultural workers. The primary infection route is via inhalation of sporangiospores or conidia that adhere to the lungs, which leads most commonly to pulmonary and rhino-orbital–cerebral infections (8, 9). When infections spread to the central nervous system, the resulting fungal meningitis caused by either pathogen is essentially fatal. Commonly prescribed antifungals to treat these infections, including amphotericin B (AmB) that is most frequently loaded into liposomes (e.g., AmBisome), and echinocandins or high doses of azoles (10, 11). Surgical debridement of the infected tissue prior to antifungal drug therapy significantly improves the outcomes of patients with mucormycosis (12, 13). Hence, more effective antifungal therapeutics are urgently needed for both diseases.

There is a stochastic body of evidence showing that fungi have sialoglycans on their surfaces that appear to contribute to pathogenicity (14). Moreover, *R. delemar* and *A. fumigatus* encode glycosyltransferases that are predicted to add sialic acid to protein targets. We hoped to learn more about fungal sialoglycans by making use of the sialic acid Ig-like lectins (Siglecs), a class of pathogen receptors located in the human host membrane. Previously, we have shown that AmB-loaded DectiSomes coated with C-type lectin receptors (CTLs), such as the Dectins, bound and killed both *A. fumigatus* and *R. delemar* (15). Herein, we employed a similar strategy and prepared AmB-loaded liposomes coated with Siglec-3 (SIG3 and *CD33*) and Siglec-15 (SIG15, CD33L3, and *SIGLEC15*) instead of CTLs to characterize sialoglycan structures expressed by these two pathogens. Siglecs (SIGs, sialic acid-binding Ig-like lectins) are host pathogen receptors that recognize diverse sialylated glycans. Fungal sialoglycans have been found primarily as sialo-glycoproteins with N-linked sialoglycans, although there is some evidence for O-linked sialoglycans. The V-set Ig-like domains of SIG3 and SIG15 recognize both distinct and overlapping sialoglycan variants (16–19), as summarized in Table ST1. SIG3 has a preference for N-linked structures and SIG15 a preference for O-linked structures. SIG15 is conserved from fish to mammals (20), whereas SIG3 emerged more recently in mammals. Sialic acid residues are nearly always located in the terminal or subterminal positions of linked sialoglycans (21) and sialo-glycoproteins that are located on the surfaces of cells or cell walls. Therefore, these two classes of fungal sialoglycans are likely to be physically exposed for binding by Siglecs.

**Fig 1:**
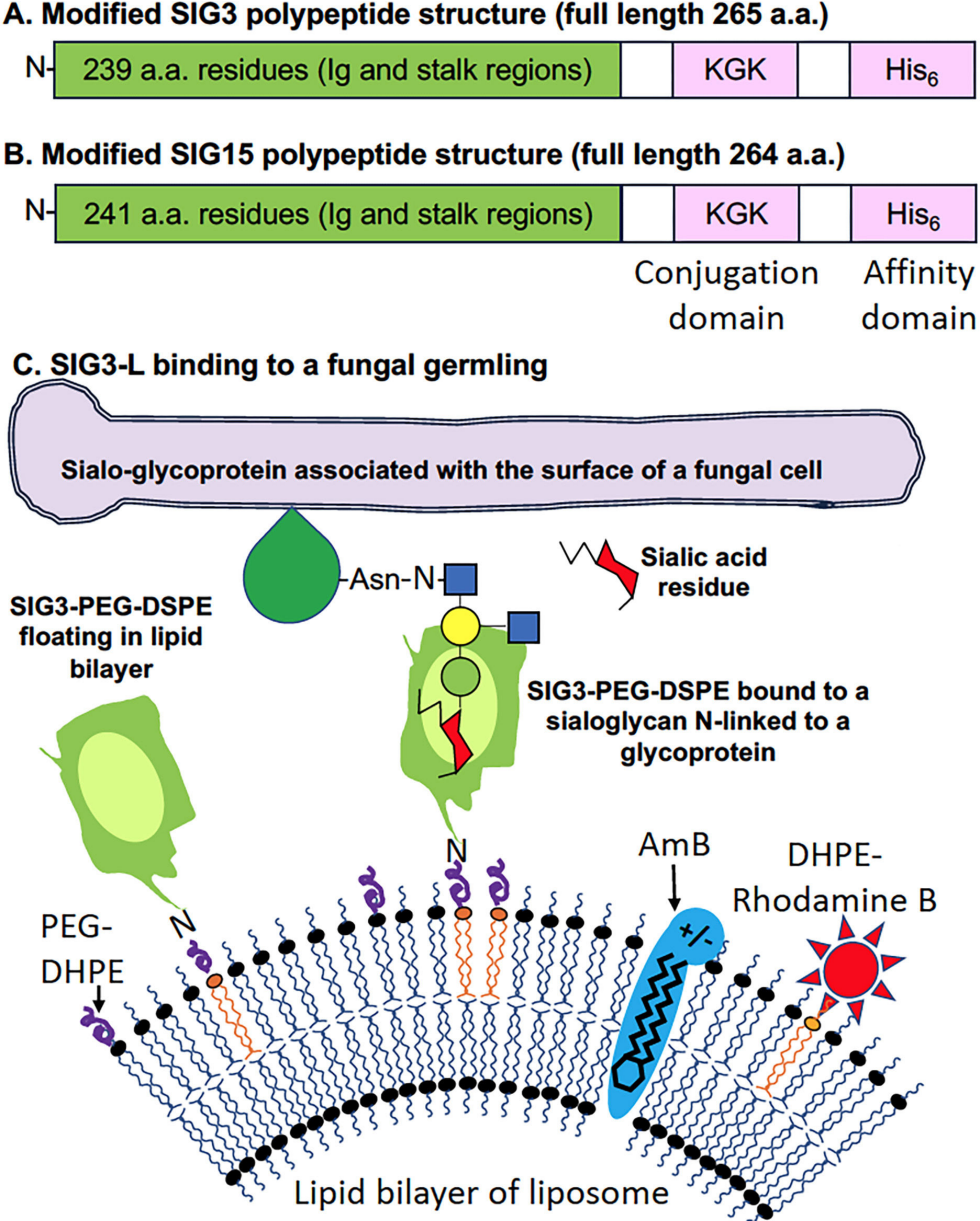
Structure of SIG3 and SIG15 polypeptides and a SIG3-targeted liposome (SIG3-L). (A and B) The structures of truncated SIG3 and SIG15 polypeptides as modified for *E. coli* expression, affinity purification, and conjugation to a lipid carrier. (C) The structure of a SIG3-targeted liposome, SIG3-L. The image shows a SIG3 polypeptide on the surface of a liposome binding to an oligoglycan with terminal sialoglycan N-linked to a glycoprotein on the surface of a fungal cell. The oval region within the green SIG3 polypeptide indicates the binding site for sialoglycans. Relative to 100 mol% liposomal lipid, SIG3-Ls contain 0.8 mol% SIG3 (~1,200 molecules/LNP), 2 mol% rhodamine B (~3,000 molecules/LNP), and 11.5 mol% AmB (~16,500 molecules/LNP). The 2D Siglec polypeptide structure shown was simplified from the published 3D crystal structure (18).

A diagram of the Siglec protein structures employed and a SIG3 pathogen receptor-targeted liposome (SIG3-L) loaded with AmB binding to a sialo-glycoprotein on a fungal cell wall is shown in Fig. 1. Compared to soluble proteins, the basic structure of AmB-loaded liposomes offers several advantages to exploring the binding of Siglec to fungal cells and their potential in targeted drug delivery to enhance antifungal drug activity (15). First, the presence of 1,200 SIG3 molecules floating on the liposome surface should enhance their avidity, strengthening their binding to closely spaced sialoglycan ligands on the fungal cell surface, over what may be achieved by the binding of a single fluorescent Siglec protein (Acro Biosystems, Cat.# CD3-HP2H7). Second, the presence of 3,000 rhodamine B fluorophores on each liposome dramatically enhances the fluorescence signal emitted by each bound liposome (15). Third, each liposome contains approximately 16,500 molecules of amphotericin B loaded in the liposomal membrane, similar to the well-characterized commercial antifungal drug AmBisome, and therefore enables assays of fungal killing (15).

Here, we employed SIG3-Ls and SIG15-Ls to study the sialoglycans of *R. delemar* and *A. fumigatus*. We obtained novel and exciting results, particularly the fact that the fluorescently tagged liposomes bound in a continuous layer on the surface of the fungal cell walls of both species during multiple fungal developmental stages. A population of sialo-glycoproteins was detected on Western blots. AmB-loaded SIG3-Ls and SIG15-Ls significantly enhanced the killing of both species over that of untargeted AmB-loaded liposomes.

## MATERIALS AND METHODS

### Fungal cells, media, and growth conditions

*R. delemar* strain 99-880 (ATCC MYA-4621) was grown and sporulated on potato dextrose agar (PDA; ThermoFisher, Cat# 0013-01-4), and the sporangiospores were stored in PBS + 0.05% Tween at 4°C. For microscopy experiments, sporangiospores were germinated, and hyphae were grown in pH 7 RPMI (Sigma-Aldrich, Cat# R8755) +0.165 M MOPS (Sigma-Aldrich, Cat# M1254) +1.5% agar plates (100,000 to 1,000,000 cells per plate) (22). Seven-millimeter-diameter plugs were transferred to a 24-well plate for staining. For killing studies, cells were grown in the liquid media in 96-well microtiter plates. *A. fumigatus* conidia (strain CEA10) were stored in sterilized deionized water at 4°C and diluted to 100,000 to 1,000,000 cells/mL in 1× Vogel’s salts + 1% glucose + 100 μg/mL kanamycin and ampicillin. Conidia were germinated and grown at 37°C either on plastic tissue culture microtiter plates or on agar plates for 8 to 14 hours for the late germling stage, when the germlings were several times longer than the diameter of the conidia.

### Siglec structure, purification, and liposome constructs

Figure 1A and B show the overall design of SIG3 and SIG15 polypeptides that were employed, and Fig. 1C is a model of a SIG3-targeted liposome (SIG3-L). The amino acid sequences, molecular weights, PIs, aliphatic index, and predicted A_280_ extinction coefficients of the two modified Siglec polypeptides and the *E. coli* codon-optimized DNA-encoding sequences are given in Fig. SF1. As native polypeptide sequences, SIG3 and SIG15 have their V-region Ig-like C2 ligand-binding domains linked to a single stalk region located at the N-terminal end to the membrane-spanning and signaling domains. Most of the other human Siglecs have more complex and repetitive stalk structures (23, 24). We focused on SIG3 and SIG15 because we were concerned that the longer repetitive stalk regions in other Siglecs might complicate the purification of functional proteins and/or their activities once loaded into a liposome. The endogenous *SIG3* gene can express a transcript-encoding isoform lacking the Ig-like V-region (25), an isoform that is not relevant to our designing a polypeptide with glycan-binding properties. The coding sequences of the two truncated Siglecs were synthesized by GenScript (USA, Piscataway, NJ) flanked with a 5´*Nco*I (CCATGG) site with an in-frame ATG start codon and a 3´*Pac*I (TTAATTAA) site with an in-frame TAA stop codon. The stop codon is preceded by a Lys–Gly–Lys conjugation site and a His_6_ tag each preceded by Gly–Ser–Gly flexible linkers. The sequences were subcloned via these two restriction endonuclease sites into the plasmid expression vector pET45b + under control of its T7 polymerase promoter. The plasmids were transformed into *E. coli* NiCo21 (DE3) cells (NEB # C2529H), a strain that has been genetically modified to produce lower levels of metal-binding proteins that might contaminate His_6_-tagged proteins when purified on nickel affinity columns. Cells were grown in 1 L of Luria broth at 37°C to an O.D. A_600_ of 0.6 and induced for T7 polymerase activity with 1 mM IPTG, followed by 4 hours of growth. Following on a six-decade-old report that antibodies can be denatured in high concentrations of guanidine hydrochloride (GuHCl) and efficiently renatured into an active form (26), we reasoned that 6M GuHCl might allow us to solubilize and later renature the related Ig-like domains of SIG3 and SIG15 after overexpression in *E. coli*. We followed a protocol that we had previously developed for the Dectins (27). The Siglec proteins were extracted directly from DPBS-washed *E. coli* cells by resuspending the cell pellets in 30 mL of a pH 8.0 lysis and binding buffer (6 M GuHCl (Fisher BioReagents; BP178), 0.1 M Na_2_HPO_4_:NaH_2_PO_4_ (1:9), 10 mM triethanolamine (Sigma-Aldrich, Cat #90279), 100 mM NaCl, 5 mM fresh 2-mercaptoethanol) by tumbling them for 1 hour at room temperature (27). After centrifugation at 10,000 × *g* for 25 minutes at 4°C, approximately 25 mL of the supernatant containing denatured proteins was recovered. The protein extract was used to resuspend 2 mL of packed nickel affinity resin (Ni-NTA Agarose, QIAGEN # 30230) and rocked gently for 1 hour at room temperature. After packing the resin in a P10 column (Millipore-Sigma, GE17-0435-01) and washing the column, the Siglec proteins were eluted in the same GuHCl buffer at pH 4.5 (27). The pH was adjusted to 7.0 with triethanolamine. The milligram yields of each protein were estimated from their theoretical molar extinction coefficients at A_280_ given in Fig. SF1. After affinity purification, approximately 20 mg of SIG3 and 40 mg of SIG15 were recovered per liter of Luria broth culture. The polypeptides were approximately 80% pure based on SDS-PAGE analysis (Fig. SF2). While still in the GuHCl buffer, the pH of a few milligrams of each protein was adjusted to pH 8.3 with triethanolamine. Bovine serum albumin (BSA, Sigma Cat # A8022) at 10 mg/mL was buffer-exchanged into 0.2 M pH 8.3 carbonate buffer to remove any trace amines. The three proteins were conjugated with a 4-molar excess of DSPE-PEG-3400-NHS (Nanosoft Polymers, 1544–3400) pre-dissolved in 1/10^th^ volume of dimethyl sulfoxide (DMSO, Millipore-Sigma Cat# D2438). Excess NHS reagent and GuHCl were removed from the modified Siglecs by chromatography on a P10 column packed with 5 mL of Bio-Gel P-6 acrylamide molecular exclusion resin (Bio-Rad Cat#150-0740) in an arginine-rich renaturation and crowding buffer RN#5 (pH 8.0, 1 M L-arginine, 0.1 M NaH_2_PO_4_, 10 mM triethanolamine, 100 mM NaCl, 5 mM EDTA, and 1 mM fresh 2-mercaptoethanol) (28) to prepare SIG3-PEG-DSPE, SIG15-PEG-DSPE, and BSA-PEG-DSPE. We were concerned that the extremely high aliphatic indices and high PIs of the two Siglecs (Fig. SF1) would make them unstable and cause irreversible protein aggregation and precipitation in physiological buffers. High concentrations of arginine are known to prevent protein aggregation and precipitation and begin the process of renaturation for some otherwise unstable proteins (29–31).

The Siglec proteins prepared from *E. coli* will not be glycosylated, and they migrated on SDS-PAGE at their expected native unmodified MWs (Fig. SF2). When made in human cells, the proteins migrate on SDS-PAGE with MWs that are much higher than that of the native polypeptides due to glycosylation. Glycosylation of SIG15 was shown to alter its interaction with T cells, but not its sialoglycan ligand specificity; hence, we expected our un-glycosylated proteins to retain their binding activities (18).

AmB was remotely loaded into commercial liposomes (FormuMax F20203A) to 11 mol% relative to moles of liposomal lipid to prepare AmB-loaded liposomes (27). Each 100-nm liposome is estimated to contain 16,500 molecules of AmB and 150,000 molecules of lipids (15). Hence, when we treat cells with liposomes delivering 1.0 umole of AmB, the parent liposomes delivered 9 umoles of lipid. Except for their surface pegylation, they are chemical and structural analogs of commercial AmBisome. SIG3-PEG-DSPE, SIG15-PEG-DSPE, and BSA-PEG-DSPE were remotely loaded into AmB-loaded liposomes via their DSPE lipid moiety at 0.8, 0.8, and 0.33 mol percent to make SIG3-Ls, SIG15-Ls, and BSA-Ls, respectively (27). BSA was loaded into the liposomes at a lower mol % ratio to account for its 2.3-fold larger molecular size of 66 kDa. Hence, by weight, BSA-Ls have the same amount of protein on their surfaces as the Siglec-coated reagents. All liposome preparations were simultaneously remotely loaded with 2 mol% DHPE-rhodamine B (27). The final concentrations of AmB and protein in typical preparations were 0.96 mg AmB/mL and 2.2 mg protein/mL, respectively. The final refolding of the Siglec polypeptides is accomplished when the liposomes in this arginine-rich buffer were diluted several hundred-fold into liposome dilution buffer 2 (LDB2) blocking and staining buffer (20mM HEPES, 10mM triethanolamine, 150mM NaCl, 10mM CaCl_2_, pH 8.0, 5% BSA, and 1mM BME freshly added) used for binding studies or when dialyzed into TAS2 buffer (20mM Tris, 13mM acetate, pH 7.5, 9% sucrose, and 1mM beta-mercaptoethanol) for cell killing studies.

We estimated the density of the Siglecs polypeptides on the liposomal surface. The surface area of a spherical 100 nm-diameter liposome may be estimated as 4πr^2^ = 31,400 nm^2^. The extracellular domain and stalk region of Siglec-3 and Siglec-15 are about the same size (Fig. SF1). From the crystal structure of this portion of Siglec-15 (18), we estimated that the Stokes radius (32) of the Siglec polypeptides we employed was ~2.6 nm. Hence, if we assume this portion of the Siglecs is roughly spherical, their area presented to the liposome surface was estimated as πr^2^ = 17.2 nm^2^. Based on this size, we estimated approximately 1,800 Siglec polypeptide molecules could potentially fit on the surface of one liposome. Hence, to accommodate their free movement on the surface of a liposome, we loaded approximately 1,200 Siglec monomers per liposome and presumed that the surface was still relatively crowded with Siglec polypeptides. We assumed a high density of Siglecs would enhance their avidity for target ligands.

### Liposome binding to fungal cells

After *R. delemar* and *A. fumigatus* had grown to the desired developmental stage on agar plates, agar plugs were removed, fixed in 6% formalin in PBS for 1 hour, and washed and treated as described previously (22). *R. delemar* binds tightly to agar during swelling of the sporangiospores, but all developmental stages stick poorly to plastic or glass. Fixed cells were treated with LDB2 blocking and liposome dilution buffer for 60 minutes. Liposomes were diluted in LDB2 such that they were delivering Siglec protein or BSA at 1 µg/100 µL (1:100 wt/vol) and stained for 60 minutes. AmB liposomes lacking any protein (Ls) were diluted equivalently. Stained cells were given four washes with LDB2. The agar plugs were mounted on microscope slides and fitted with a coverslip. Photographic images were taken on an RVSF1000 ECHO Revolve R4 microscope (VWR International, LLC) top–down for agar plugs. Separate brightfield images, TXRED (filters Ex560/Em630) images, and merged Z-overlay images were recorded digitally using the microscope’s software. The pixel area per image of red fluorescence from multiple images was estimated using a Cell Profiler subroutine, AreaPipe developed for this purpose (33).

### Neu5Ac specificity of Siglec liposomal binding

Siglecs bind primarily to sialic acid residue- (Neu5Ac-) containing oligoglycans. However, lymphoid reporter cell lines expressing Siglec-5 and Siglec-14 were activated by lipids extracted from the Ascomycete *Trichophyton* spp. and by purified long-chain alkanes lacking Neu5Ac residues (34). To prove that SIG3-L and SIG15-L recognition of fungi was sialoglycan-specific, we assayed binding after removing sialoglycans with Sialidase AU from *Arthrobacter ureafaciens* and N-linked sialoglycans with PNGase F from *Flavobacterium meningosepticum*. The enzyme stocks were purified as described previously (35, 36) and aliquoted in a buffer containing 20 mM HEPES, 100 mM NaCl, pH 7, 10% glycerol and stored at −80°C. Thirty microliters containing 3 µg of each enzyme was dripped onto *R. delemar* cells on the surface of 300 µL agar plugs such that the final concentration after diffusion was 1:100 wt/vol. The plugs were incubated for 1 hour at 37°C and then overnight at room temperature. Rhodamine red fluorescent SIG3-L and SIG15-L binding was assessed relative to a buffer-treated control by taking multiple top–down microscope images of fungal cell fluorescence.

### Liposome killing studies on fungal cells

Siglec-targeted AmB-loaded liposome inhibition and killing assays on *R. delemar* were conducted in 96-well microtiter plates without changing the media after the initial plating of cells because their hyphae do not stick to plastic plates, but they do stick to micropipette tips and may be accidentally removed from the wells (22). Sporangiospores were plated (800/well) in 90 µL of pH 7 RPMI +0.165 M MOPS and grown for 2 hours at 37°C. Reagent liposomes delivering various concentrations of AmB were added in 10-µL aliquots per well and incubated at 37°C with shaking overnight. Sixteen to 24 hours later, 20 µL of CellTiter-Blue (CTB, Promega; Cat.# G8081; Madison, WI, USA) resazurin reagent was added to each well and incubated at 37°C according to the manufacturer’s instructions. The pink fluorescence of the reduced product resorufin was quantified (Ex485/Em590) in an Agilent BioTek Synergy H1 fluorescent microplate reader after 60 minutes of incubation with the reagent. The background fluorescence in wells with all reagents, but lacking cells, was subtracted. *A. fumigatus* was plated at 4,500 conidia per well in 96-well microtiter plates, treated immediately with liposomes, and grown overnight. The old medium was removed, and metabolic activity was assayed 4 hours after the addition of CTB reagent diluted into fresh media (27).

### Western blot analysis

To prepare Siglec proteins to probe on Western blots, purified His_6_-tagged SIG3 and SIG15 in GuHCl storage buffer were buffer-exchanged first into RN#5 crowding buffer and then into LDB2. Western blots were probed with the Siglec proteins diluted to 1 μg/100 μL (1:100wt/vol) in LDB2.

To prepare fungal sialo-glycoproteins for Western blots, *R. delemar* (Rd) and *A. fumigatus* (Af) were grown in liquid formulations of the same media described above with shaking at 37°C for 2 days, when the solutions were dense with hyphae. Hyphae were collected by gravity on a 40-micron Nylon Mesh cell strainer (Fisher #22363547) and washed once briefly with 2 mL of HBS (50 mM HEPES, pH 8.0 with 150 mM sodium chloride and fresh protease inhibitor cocktail, Roche, Complete Mini, EDTA free #1183617001). Proteins were extracted into 2% of the media volume of HBS by homogenizing with a Dounce homogenizer for 5 minutes and determining the flow-through on another 40-micron filter to prepare the RdHBS and AfHBS protein samples. The residual hyphae were again extracted with the homogenizer for 5 minutes, but with RIPA detergent buffer (50 mM HEPES, pH 8.0, with 150 mM sodium chloride, 1.0% Igepal CA-630 (NP-40), 0.5% sodium deoxycholate, and 0.1% sodium dodecyl sulfate and fresh protease inhibitor), to prepare RdRIPA and AfRIPA samples. Thirty microgram samples of proteins diluted into 2× SDS-Laemmli sample buffer (37) were heated to 90°C for 5 minutes, resolved on a precast 12% Acrylamide SDS PAGE gel (Bio-Rad #4561093), and blotted to a PVDF membrane (Bio-Rad TransBlot Turbo #BR20240603). The membrane was incubated overnight with anti-His_6_-tagged monoclonal antibody HRP conjugate (Invitrogen #MA1-21315-HRP) diluted 1:500 wt:vol. The membranes were incubated with ECL reagents (Thermo Supersignal West Pico Stable Luminol Peroxide Substrate #1862123) and exposed on a Bio-Rad ChemDoc Imaging System for 30 to 60 seconds. We found the more sensitive ECL reagent kit (Thermo SuperSignal West Femto Maximum Sensitivity Substrate #34095) was too sensitive for our blots, causing the various bands to merge together even with 1 second exposures.

### Statistics and graphics

The quantitative binding data from AreaPipe are in .csv files, which were processed in Microsoft Excel (version 16.76) and moved into GraphPad Prism version 10.0.2, where scatter bar plots were prepared. Cell killing data are stored as Excel files and plotted for presentation in GraphPad Prism. *P*-values were estimated using the Student’s *t*-Test (T.Test) in Excel. When the data from one or both members of a comparison were nonparametric (not normally distributed), Mann–Whitney (P_MW_) values were estimated in Prism. Prism recommends not reporting P_MW_ values as less than 0.0001. Pixel density scans of photographic images of SDS-PAGE gels were performed in ImageJ (Version 1.53 a).

## RESULTS

### Preparation of Siglec-targeted liposomes

We cloned the Ig-like domains and stalk regions of SIG3 and SIG15, expressed the encoded polypeptides in *E. coli,* and used a novel protocol detailed in the method section to purify them in a denatured state in 6 M GuHCl. Both proteins were conjugated to DSPE-PEG and then partially renatured in 1 M arginine crowding buffer. We coated AmB-loaded, rhodamine B-tagged liposomes with SIG3, SIG15, and BSA to prepare SIG3-L and SIG15-L liposomes and BSA-Ls as a protein-coated control. The model of a SIG3-L (Fig. 1C) shows that the SIG3–PEG–DSPE conjugate is free to float on the surface of the liposome membrane via its DSPE lipid moiety. SIG3 extends from the surface of the liposome via its own flexible neck region and a 3,400 Da PEG linker. Siglec polypeptides may need to move and rotate freely to bind to their cognate sialo-glycoproteins, which might be presented in different orientations and spacings on a fungal cell.

### Binding to *R. delemar*

**Fig 2:**
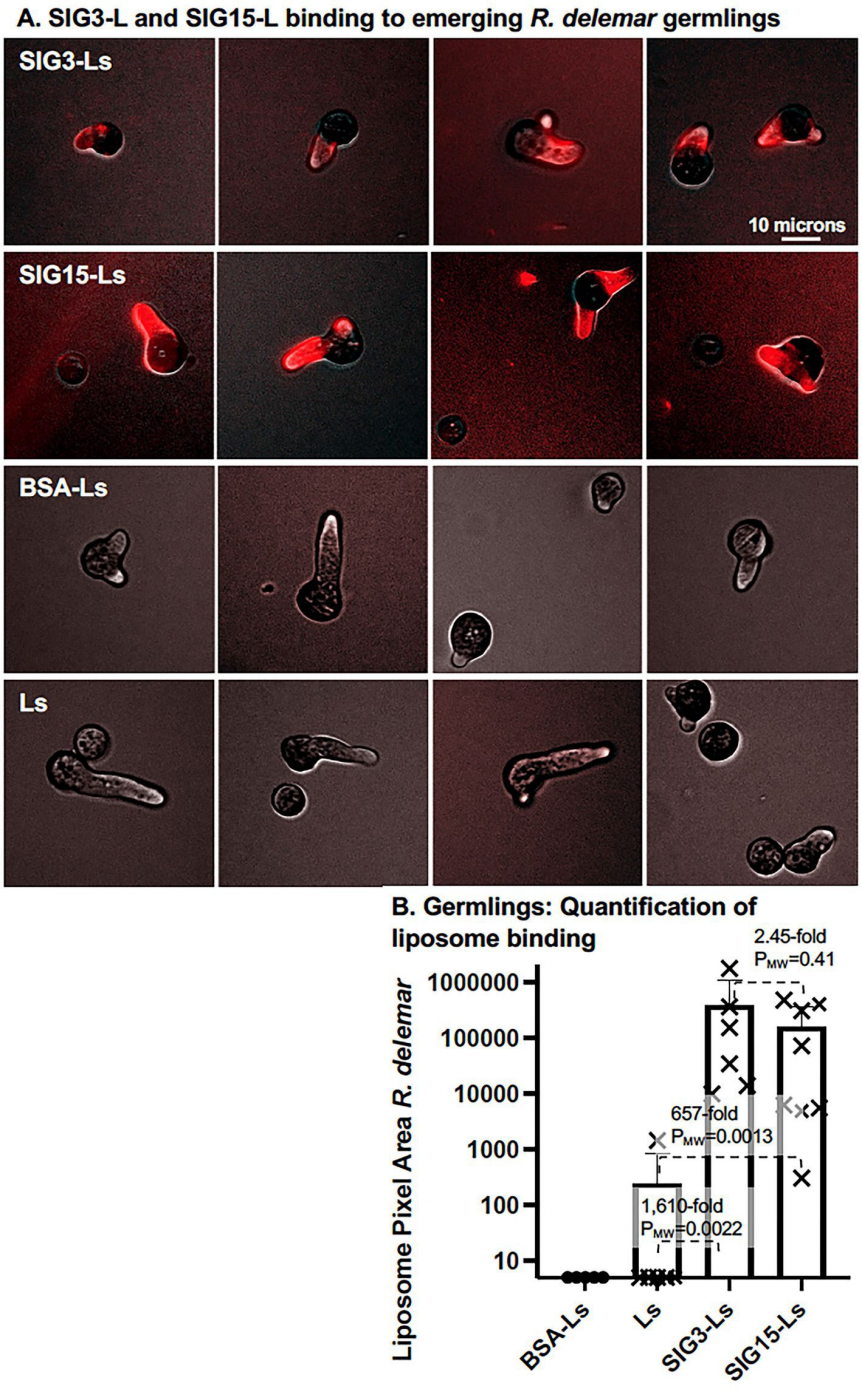
Binding of SIG3-Ls and SIG15-Ls to newly emerging *R. delemar* germlings. (**A**) *R. delemar* sporangiospores were germinated on agar plates for 3 hours. Agar plugs were removed, fixed, stained with rhodamine B-tagged SIG3-Ls, SIG15-Ls, and BSA-Ls delivering 1 μg/100 μL (1:100 wt/vol) protein and similarly diluted with plain Ls, and photographed top–down at 20× (0.8 NA lens) with combined red fluorescence and weak brightfield imaging to outline cells. Images were equivalently cropped and enhanced for total brightness. A size bar indicates the degree of magnification. (**B**) A scatter bar plot quantifies the areas of red fluorescence from multiple random images like those in A (*N* = 6). Fold differences and Mann–Whitney *P*-values as compared to binding by Ls are indicated. Bars and whisker plots indicate standard errors from the mean.

*R. delemar* sporangiospores were germinated for 3 hours on agar plates. By this time, spores were swollen with emerging germlings. Agar plugs were removed, fixed, and prepared for Siglec-targeted liposome binding assays. The stained fungal cells on the surface of the agar were photographed. Rhodamine red fluorescent SIG3-Ls and SIG15-Ls bound strongly to the emerging germling but not visibly to the swollen sporangiospore (Fig. 2A). Binding appeared to be specific to the cell wall or immediately adjacent to it. This is in contrast to the Dectin targeting of *R. delemar* (22, 33), where DectiSomes bound to the secreted exopolysaccharide (EPS). As expected, we did not detect any obvious binding with untargeted control Ls or BSA-Ls. A log_10_ scatter bar plot (Fig. 2B) quantified the area of red fluorescent binding from several independent images. SIG3-Ls and SIG15-Ls bound 1,610-fold (P_MW_ = 0.0022) and 657-fold (P_MW_ = 0.0013) more efficiently to *R. delemar* germlings than control Ls, respectively. The extent of SIG3-L binding was always greater than that for SIG15-Ls, but the differences were not always statistically significant.

**Fig 3:**
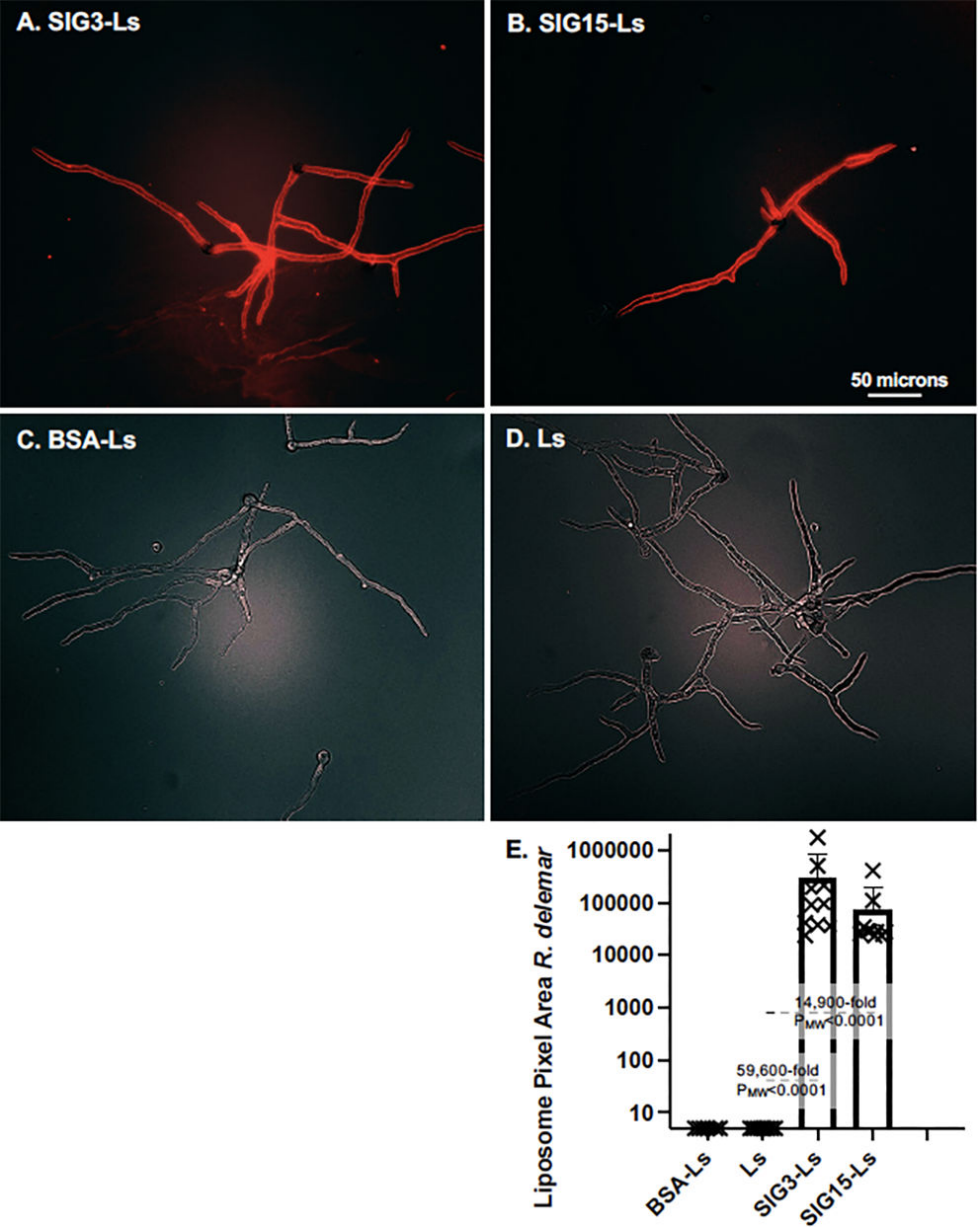
Binding of SIG3-Ls and SIG15-Ls to *R. delemar* hyphae. *R. delemar* sporangiospores were germinated on agar plates for 7 hours. Agar plugs were removed, fixed, stained with rhodamine B-tagged (**A**) SIG3-Ls, (**B**) SIG15-Ls, and (**C**) BSA-Ls delivering 1 μg/100 μL (wt/vol) proteins, and (**D**) plain Ls similarly diluted and photographed top–down at 20× (0.8 NA lens) with combined red fluorescence and weak brightfield imaging to outline cells. Images were enhanced for total brightness. A size bar indicates the degree of magnification. (**E**) A scatter bar plot quantifies the areas of red fluorescence from multiple random images like those in A–D (*N* = 10). Fold differences and Mann–Whitney *P*-values as compared to binding by Ls are indicated. Bars and whisker plots indicate standard errors from the mean.

Encouraged by these observations, we examined the staining of young hyphal colonies that had developed after sporangiospores were germinated on agar for 7 hours (Fig. 3). SIG3-Ls and SIG15-Ls bound strongly all along the cell wall of hyphae (Fig. 3A and B). Binding was essentially equivalent between the older subapical part of the hypha and the younger rapidly growing hyphal tip. Again, Siglec-targeted liposomes did not bind efficiently to the parent sporangiospores. Binding of untargeted control liposomes, BSA-Ls and Ls, to sporangiospores or hyphae was below our detection limit (Fig. 3C and D). We quantified the relative area of fluorescent liposome binding from multiple randomly taken images (Fig. 3E). SIG3-Ls and SIG15-Ls bound 60,000-fold (P_MW_ <0.0001) and 15,000-fold (P_MW_ <0.0001) more efficiently to *R. delemar* hyphae than control Ls, respectively. Again, SIG3-Ls bound more strongly than SIG15-Ls. SIG3-Ls and SIG15-Ls bound similarly along all the cell wall surfaces of larger hyphal colonies (Fig. SF3).

### Binding to *A. fumigatus*

**Fig 4:**
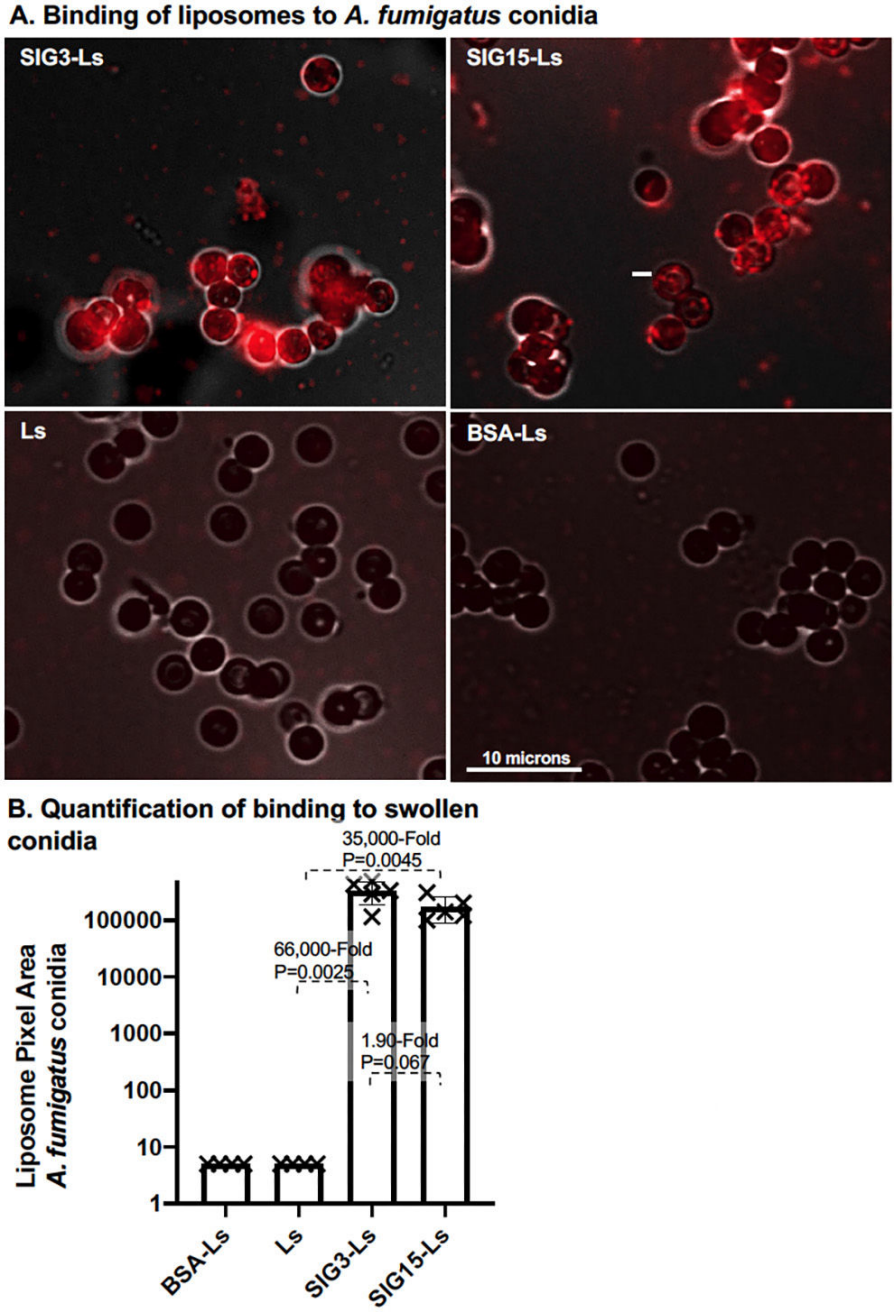
Binding of SIG3-Ls and SIG15-Ls to *A. fumigatus* swollen conidia. (**A**) Binding to conidia. *A. fumigatus* conidia were swollen on agar plates for 8 hours. Agar plugs were removed, fixed, stained with rhodamine B-tagged SIG3-Ls, SIG15-Ls, BSA-Ls, and plain Ls, and photographed top–down at 60× (1.3 NA lens) with combined red fluorescence and weak brightfield imaging to outline cells. Images were equivalently cropped and enhanced for total brightness. A size bar indicates the degree of magnification. (**B**) Quantification of binding to conidia (*N* = 6). A scatter bar plot shows the amount of red fluorescence, as quantified using a CellProfiler subroutine AreaPipe. Fold differences and *P*-values show the differences from the untargeted control liposomes, Ls.

*A. fumigatus* conidia were germinated for 8 hours on agar. We found that SIG3-Ls and SIG15-Ls bound to nearly all swollen conidia of *A. fumigatus* (Fig. 4A), in contrast to the lack of efficient binding to swollen sporangiospores of *R. delemar*. As expected, untargeted control liposomes, Ls and BSA-Ls, did not bind detectably. SIG3-Ls and SIG15-Ls bound 66,000-fold (*P* = 0.0025) and 35,000-fold (*P* = 0.0045), respectively, more strongly than Ls (Fig. 4B).

**Fig 5:**
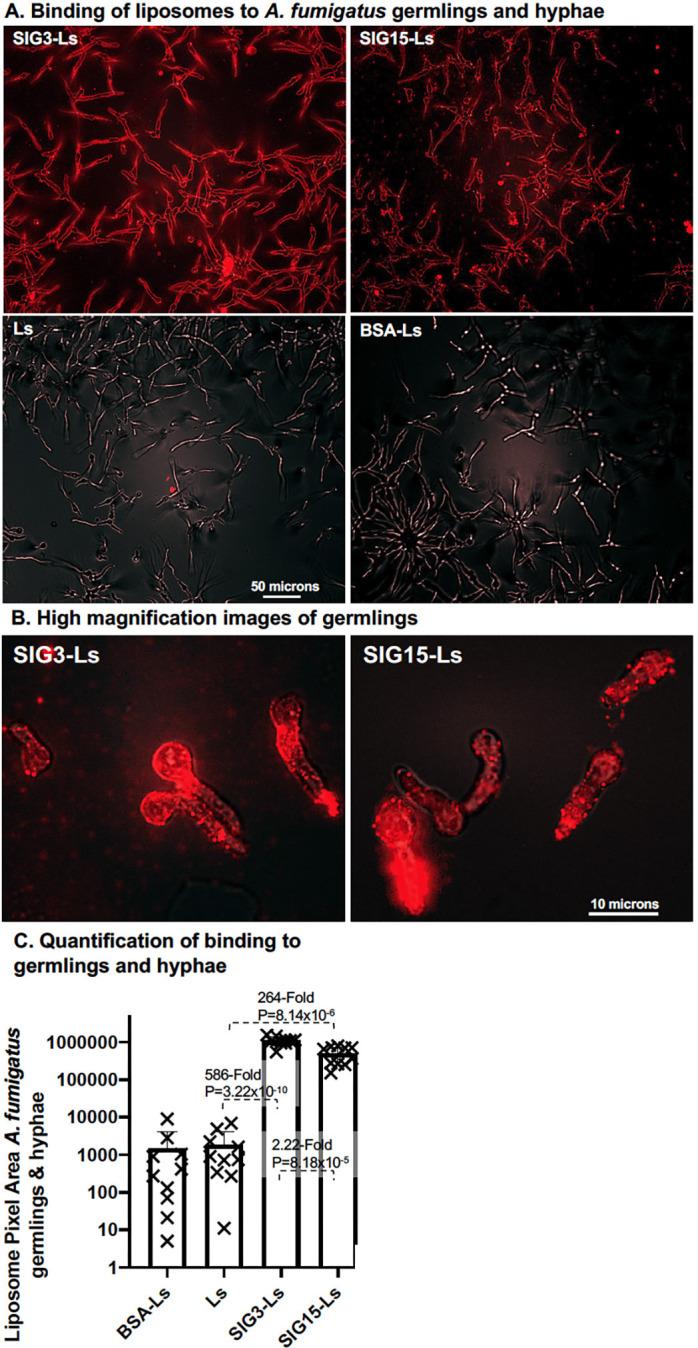
Binding of SIG3-Ls and SIG15-Ls to *A. fumigatus* germlings and hyphae. (**A**) *A. fumigatus* conidia were germinated on agar plates for 14 hours. Agar plugs were removed, fixed, stained with rhodamine B-tagged SIG3-Ls, SIG15-Ls, BSA-Ls delivering 1 μg/100 μL proteins, and plain Ls, and photographed top–down at 20× (0.8 NA lens) with combined red fluorescence and weak brightfield imaging to outline cells. A size bar indicates the degree of magnification. (**B**) Higher-magnification images of early-stage germlings show that much of the staining is tightly associated with the spore and germ tube. A size bar indicates the degree of magnification. (**C**) A scatter bar plot shows the amount of red fluorescence in multiple images (*N* = 10). Fold differences and *P*-values show the differences from the untargeted control liposomes, Ls.

To examine if SIG3-Ls and SIG15-Ls can bind to *A. fumigatus* germlings and hyphae, we let the conidia grow on minimal agar plates for 14 hours and stained with the liposomes (Fig. 5A). SIG3-Ls and SIG15-Ls bound strongly and specifically over the entire cell wall of conidia, germlings, and elongated hyphae, while control liposomes did not bind detectably. For some hyphal tips that curl upward off the agar surface and out of the focal plane, binding was not observed. SIG3-Ls and SIG15-Ls bound 586-fold (*P* = 3.22 × 10^−10^) and 264-fold (*P* = 8.14 × 10^−6^) more strongly than Ls, respectively (Fig. 5C). Thus, SIG3-Ls bound 2.2-fold (*P* = 8.2 × 10^−5^) more strongly than SIG15-Ls. Higher-magnification images suggest that most of the binding is to the cell walls of both *A. fumigatus* conidia and hyphae (Fig. 5B).

### Neu5Ac specificity of binding

**Fig 6:**
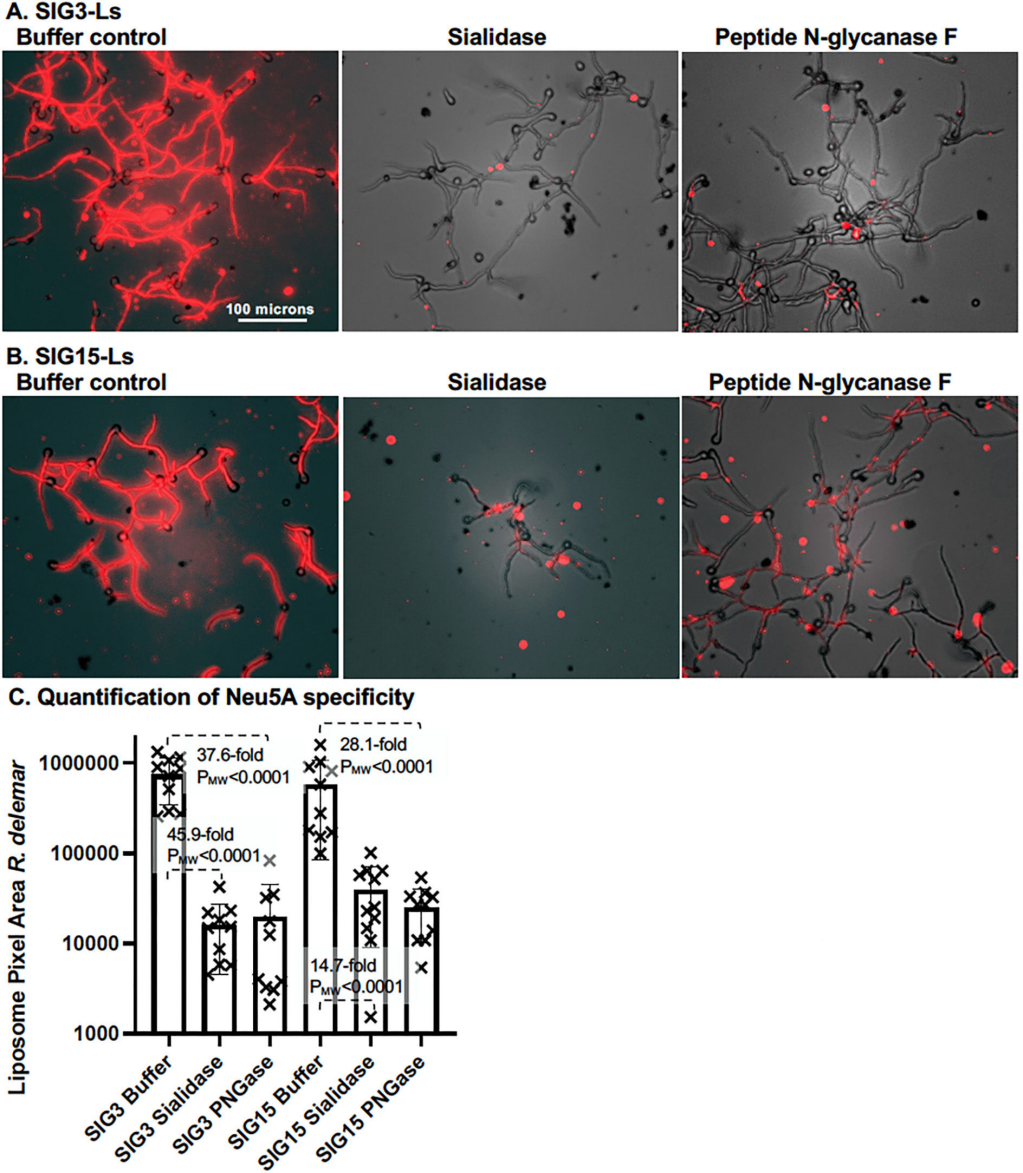
Neu5Ac and sialo-glycoprotein specificity of SIG3-L and SIG15-L binding to *R. delemar. R. delemar* was germinated and grown on agar for 7 hours. Agar plugs (300 μL volume) were fixed, washed, and treated overnight with 3 μg/30 μL of sialidase, 3 μg/30 μL of peptide N-glycanase F, or buffer as a control. Hence, the final concentration of the enzymes after diffusion into the agar plugs was 1 μg/100 μL (wt/vol). Cells were photographed top–down through a coverslip at 20× (NA 0.4 lens). Images were enhanced equivalently for red fluorescence and brightness. (**A**) SIG3-L binding. (**B**) SIG15 binding. (**C**) A scatter bar plot quantifies the binding from multiple images for each treatment (*N* = 10). Fold differences and P_MW_-values are compared to those of the buffer-treated controls.

To confirm that SIG3-L and SIG15-L recognition of fungal cells was sialoglycan-specific, we assayed binding to *R. delemar* hyphae after removing Neu5Ac with sialidase AU and N-linked glycans from proteins with PNGase F (Fig. 6). Sialidase AU cleaves terminal sialic acid residues from oligosaccharides and glycoproteins (35) and has the potential to prevent binding by both Siglecs. PNGase F is an amidase that cleaves between the innermost GlcNAc and asparagine residues on N-linked glycoproteins (36). PNGase F cleavage was expected to prevent SIG3 binding. It may also prevent SIG15 binding if SIG15-Ls also bound to N-linked sialoglycans. *R. delemar* hyphae were grown on agar plugs, fixed, treated with each enzyme overnight, and assayed for SIG3-L and SIG15-L binding relative to buffer-treated controls. We found that sialidase and PNGase F treatment both dramatically reduced the binding by SIG3-Ls and SIG15-Ls (Fig. 6A and B). We quantified the binding from multiple fluorescent images. Treatment with either enzyme caused at least an order of magnitude decrease in binding by SIG3-Ls and SIG15-L (Fig. 6C, P_MW_ <0.0001). These results strongly suggest that both reagents bind primarily to sialoglycans N-linked to sialo-glycoproteins.

### SIG3 and SIG15 proteins bind fungal sialo-glycoproteins on Western blots

**Fig 7:**
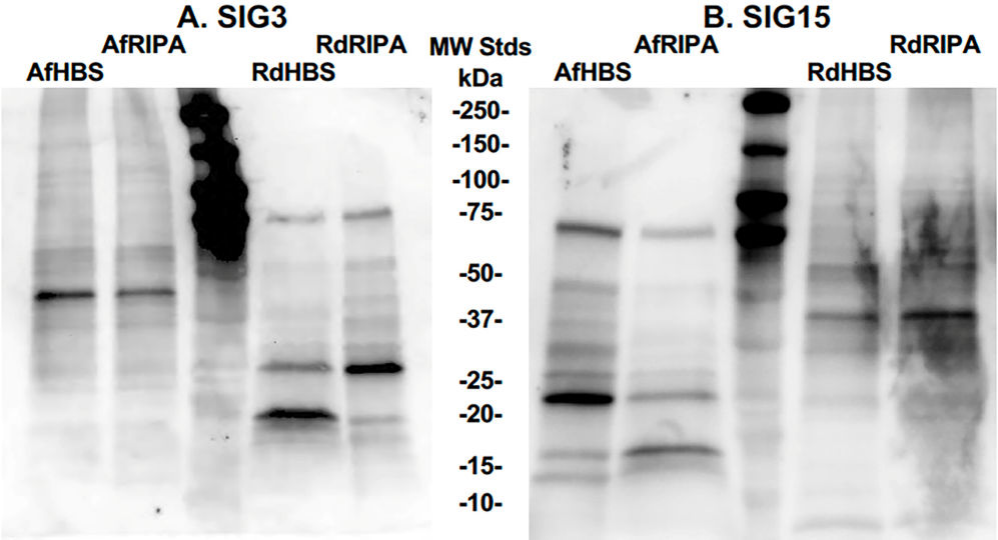
Western blots of sialo-glycoproteins probed with SIG3 and SIG15 proteins. The hyphae of both species, Af and Rd, were extracted with ionic HBS buffer and detergent RIPA buffer to prepare RdHBS, AfHBS, RdRIPA, and AfRIPA protein samples. Protein samples of 30 μg were resolved by SDS-PAGE and blotted to a membrane. Protein blots were incubated with His_6_-tagged SIG3 protein (**A**) and SIG15 protein (**B**) and then anti-His-tag antibody conjugated with HRP that generated chemiluminescent signals for protein bands. Molecular weight standards (MW Stds) were run in the central lane of the blots, and their sizes are indicated.

*R. delemar* (Rd) and *A. fumigatus* (Af) proteins were extracted from hyphae into an ionic HBS buffer and a detergent RIPA buffer. RdHBS, AfHBS, RdRIPA, and AfRIPA protein extracts were resolved by SDS PAGE. Protein blots were incubated with His_6_-tagged SIG3 and SIG15 proteins. Anti-His-tag antibody conjugated with HRP was used to detect the Siglecs bound to hyphal proteins. SIG3 and SIG15 each bound to numerous bands of proteins in both the ionic HBS buffer and detergent RIPA buffer extracts from both species (Fig. 7). Most of the protein bands detected were between 15 kDa and 75 kDa. On average, the *A. fumigatus* proteins detected were larger than those from *R. delemar*. These results strongly support our evidence that SIG3 and SIG15 bind fungal sialo-glycoproteins.

### Killing fungal cells

**Fig 8:**
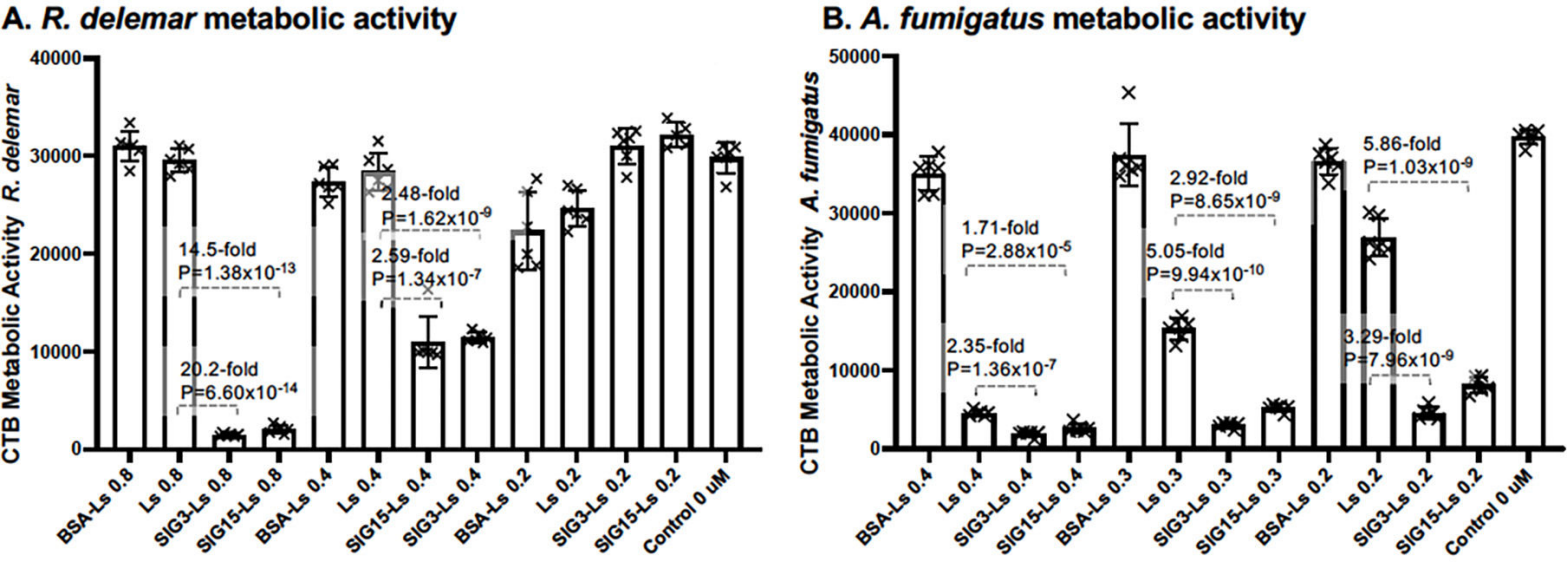
Inhibition and killing of *R. delemar* and *A. fumigatus* by Siglec-targeted liposomes. *R. delemar* sporangiospores (**A**) and *A. fumigatus* conidia (**B**) were germinated in 96-well microtiter plates and assayed for viability after adding SIG3-Ls and SIG15-Ls delivering various concentrations of AmB from 0.2 to 0.8 μM. Reduced live cell metabolic activity was measured using the REDOX-sensitive reagent CellTiter-Blue (CTB). The data are quantified in scatter bar plots (*N* = 6 wells for each bar). Fold differences and *P*-values compared CTB activity levels to that of untargeted Ls. Bars and whisker plots indicate standard errors from the mean.

*R. delemar* sporangiospores were incubated for 2 hours when most of the sporangiospores had just started to swell but before germ tubes emerged. SIG3-Ls, SIG15-Ls, BSA-Ls, and Ls delivering a final concentration of 0.8, 0.4, and 0.2 µM AmB, respectively, were added to each well. After an overnight incubation, the residual metabolic activity was assayed with CellTiter-Blue (CTB) reagent (Fig. 8A). CTB measures live cell mitochondrial-dependent REDOX activity. SIG3-Ls and SIG15-Ls delivering 0.8 µM AmB reduced the metabolic activity by 20.2-fold (*P* = 6.6 × 10^−14^) and 14.5-fold (*P* = 1.38 × 10^−13^), respectively, relative to untargeted AmB-loaded Ls. SIG3-Ls and SIG15-Ls delivering 0.4 µM AmB reduced the metabolic activity by 2.5-fold (*P* = 1.62 × 10^−9^) and 2.6-fold (*P* = 1.34 × 10^−7^), respectively, relative to untargeted Ls.

*A. fumigatus* conidia were seeded at fivefold higher densities than for binding studies into 96-well microtiter plates than *R. delemar* sporangiospores. *A. fumigatus* cells are much smaller than those of *R. delemar*, and more biomass is required to assay the metabolic activity. Immediately after plating cells, SIG3-Ls, SIG15-Ls, BSA-Ls, and Ls delivering final concentrations of 0.4, 0.3, and 0.2 µM AmB were added to each well. After an overnight incubation, the residual live cell metabolic activity was assayed with CTB (Fig. 8B). SIG3-Ls and SIG15-Ls delivering 0.4 µM AmB reduced the metabolic activity by 2.4-fold (*P* = 1.4 × 10^−7^) and 1.7-fold (*P* = 2.9 × 10^−5^), respectively, relative to untargeted AmB-loaded Ls. SIG3-Ls and SIG15-Ls delivering 0.3 µM AmB reduced the metabolic activity by fivefold (*P* = 9.9 × 10^−10^) and 2.9-fold (*P* = 8.7 × 10^−9^), respectively, relative to untargeted Ls. SIG3-Ls and SIG15-Ls delivering 0.2 µM AmB reduced the metabolic activity by 3.3-fold (*P* = 8.0 × 10^−9^) and 5.9-fold (*P* = 1.03 × 10^−9^), respectively, relative to untargeted Ls. Hence, SIG3-L- and SIG15-L-mediated targeting of AmB was significantly more effective at killing *R. delemar* and *A. fumigatus* cells than the untargeted liposomal drugs. It is worth noting that plain Ls were effective at reducing their metabolic activity at any of these concentrations, even though the BSA-Ls were not. Perhaps, the coating of BSA interfered with the interaction of liposomal AmB with fungal ergosterol.

## DISCUSSION

It was previously shown that the sialoglycan-specific lectins (also known as agglutinins) LFA, LPA, SNA, and/or MAA (Table ST1) bound modestly well to sporangiospores and yeast cells of *Rhizopus microspores* (frequently known as *Mucor polymorphosporus*) (38, 39) and to the conidia of the Ascomycete *A. fumigatus* (40). *R. microspores* is a distant Mucormycete relative of *R. delemar*. We found SIG3-Ls and SIG15-Ls bound to the conidia, germlings, and hyphae of *A. fumigatus*. By contrast, SIG3-Ls and SIG15-Ls bound strongly to *R. delemar* germlings and hyphae, but not significantly to its sporangiospores, as did the sialoglycan-specific agglutinins to *R. microsporus*. It appears that under the conditions we used to prepare *R. delemar* sporangiospores and germinate them, there is a developmental program that upregulates sialoglycan expression during germination, and the expression is maintained during hyphal development. As to the lack of strong binding to *R. delemar* sporangiospores relative to agglutinin binding to those of its distant *Mucor* relative, this difference could be due to evolutionary differences in sialoglycan expression between the two species or to species-specific differences or to differences in the selectivity of the Siglecs we used relative to that of the agglutinins used to examine *R. microsporus*. We found no previous reports of sialoglycans being located in the cell wall of *Aspergillus* spp. hyphae, including a biochemical analysis of the *A. fumigatus* hyphal cell wall (41).

*R. delemar* and *A. fumigatus* are estimated to have diverged from a common fungal ancestor 800 million to 1.4 billion years ago (42). Furthermore, the Mucormycetes (e.g.*, R. delemar*) are one of the oldest phyla of fungi, branching off the fungal tree of life shortly after fungi diverged from a common ancestor with animals (42, 43). Considering the numerous complex biochemical steps involved in sialoglycan and sialo-glycoprotein synthesis (44), it is hard to imagine this pathway evolving independently in two such distantly related fungal lineages. Therefore, it is likely that the sialoglycan ligands of SIG3 and SIG15 detected herein will be found among many species throughout the fungal kingdom. It should be mentioned that there are 13 other members in the human Siglec family (20) that have specificities for diverse sialoglycan variants (17) and could be employed to broaden the analysis of fungal sialoglycans. Previously, Siglec-1, Siglec-14, and Siglec-15 have been associated with microbial infections (24, 45). Human polymorphisms in SIG15 are correlated with susceptibility to *Candida* vaginitis (46), and silencing of murine SIG15 results in an increase in the fungal burden of *Candida* in mouse models of vaginitis (46).

After five decades of research on sialoglycans and sialo-glycoproteins in fungi, there is still more speculation than solid understanding as to their structures, cellular distribution, and biological and biochemical functions (14, 47). In 1992, Hamilton et al. (48) isolated a sialoglycan-specific monoclonal antibody (3C2) and used it to demonstrate the presence of sialo-glycoproteins on the surfaces of both encapsulated and noncapsulated strains of *Cryptococcus neoformans*. Neuraminidase and proteases both prevented the antibody from reacting with sialoglycans linked to protein (48). In general, invertebrate and plant agglutinins (also known as hemagglutinins, lectins) that are specific for partially characterized classes of sialoglycans (Table ST1) have been used to characterize fungal sialoglycans, and not antibodies. Fluorescence cell cytometry showed that the agglutinins LFA and SNA bound to *C. neoformans* yeast cells, but not after treatment with neuraminidase (49). Neuraminidase treatment significantly increased phagocytosis of *C. neoformans* yeast cells by mouse macrophages. Sialidase treatment of *R. microsporus* dramatically reduced the molecular weight of agglutinin target proteins and significantly increased the efficiency of phagocytosis by monocytes and neutrophils (38, 39). Sialoglycans were similarly identified on the conidial surfaces of four different species and several strains of *Aspergillus* (40). Sialoglycan levels were fivefold higher on the surface of pathogenic *A. fumigatus* than the three nonpathogenic *Aspergillus* spp. Taken together, these studies suggest that, at a minimum, fungal sialoglycans support pathogenesis by promoting host tissue adhesion and reducing phagocytosis.

There is a much larger body of published data on mammalian cell sialoglycans and, particularly, their role in cancer. During the neoplastic transformation of cancer cells, there are steady increases in the sialylation of surface glycoproteins (50–53). Hyper-sialylation appears to promote metastasis and tumor initiation at some distance from the initial cancer (54). Sialylation and hyper-sialylation are also essential for reprogramming mammalian somatic cells and maintaining stem cell pluripotency (55, 56). Each fungal cell is totipotent and, therefore, a stem cell, so perhaps this relationship is not surprising.

Binding by both SIG3-Ls and SIG15-Ls appeared to be specific for the cell walls of *R. delemar* and *A. fumigatus*. This result is quite distinct from those of our previous studies with C-type lectin-targeted Dectin-1-coated DectiSomes, which primarily bound to ligands in the exopolysaccharide (EPS) matrix of *R. delemar* that were spaced from closely adjacent to several microns from the cell wall (22). Dectin-3-targeted DectiSomes also bound to the *R. delemar* EPS often spaced a few microns from the cell wall, although some binding appeared in patches on the surface of the cell wall (33). Similarly, Dectin-1- and Dectin-2-targeted liposomes bound to the EPS associated with *A. fumigatus,* not to the cell wall (27, 28). The ligands of these three Dectins are oligoglucans and oligomannans, which we expected to be in polysaccharides in the cell wall and EPS matrix, but also may have been linked to glycoproteins. The sialoglycans in fungi are reported to be linked to sialo-glycoproteins (14, 39). Our data strongly suggest the sialo-glycoprotein ligands of SIG3 and SIG15 are associated with the cell wall and not the EPS matrix, and, hence, are physically closer to cells than the EPS ligands we have identified for the Dectins. Digestion with sialidase AU and PNGase F reduced the binding of Siglec-3 and Siglec-15 to the hyphae of both *A. fumigatus* and *R. delemar* by more than 95%. These results confirm that their cognate ligands are sialo-glycoproteins. Ionic and detergent buffer extracts from the hyphae of both species contained populations of sialo-glycoproteins that bound to SIG3 and SIG15 on Western blots. These results provided further evidence that the sialo-glycans detected on the surface of the fungi were linked to sialo-glycoproteins.

These two Siglec-targeted liposomes bound to the cell wall and closer to the cells than the dectin-targeted liposomes that bound to more distant exopolysaccharide matrix surrounding the cells. Hence, it was surprising that SIG3-Ls and SIG15-Ls were less effective or no more effective than Dectin-1-, Dectin-2-, Dectin-3-, and/or DC-SIGN-targeted AmB-loaded liposomes at killing *R. delemar* and *A. fumigatus in vitro* (22, 27, 28, 33, 57, 58). The reasons for this difference are unclear.

### Conclusion

We floated the sialoglycan recognition domains of SIG3 and SIG15 in the membranes of AmB-loaded liposomes, analogs of AmBisome. SIG3-Ls and SIG15-Ls bound an order of magnitude more efficiently to the cell walls of two highly divergent fungal pathogens, *R. delemar* and *A. fumigatus*, than did untargeted liposomes. Our data show both Siglec-targeted liposomes bound in a continuous layer over the hyphal cell walls of both species. This appears to be the first such evidence that there is a layer of sialoglycans on the cell walls of any fungal species. Enzymatic removal of the sialoglycan ligand targets confirmed that the ligands of both SIG3-L and SIG15-Ls contained Neu5Ac in sialo-glycoproteins. Numerous SIG3- and SIG15-specific sialo-glycoproteins were detected in protein extracts from both species. Siglec-targeted AmB-loaded liposomes were modestly efficient at killing both fungal species. Our data suggest that cell wall sialo-glycoproteins may be broadly distributed throughout the fungal kingdom.

## Data Availability

All new data discussed are included in this article, and all information and data from outside sources are appropriately cited.

## SUPPLEMENTAL MATERIAL

Supplemental material aac.01720-24-s0001.docx Table S1; Fig. S1 and S2.

ASM does not own the copyrights to Supplemental Material that may be linked to, or accessed through, an article. The authors have granted ASM a non-exclusive, world-wide license to publish the Supplemental Material files. Please contact the corresponding author directly for reuse.
