# Supplementary material for: Siglec-targeted liposomes to identify sialoglycans present on fungal pathogens
Source: Antimicrob Agents Chemother. 2025 Mar 14;69(4):e01720-24. doi: 10.1128/aac.01720-24 (PMC11963605; doi:10.1128/aac.01720-24)
Supplement: Supplemental material — Table S1; Fig. S1 and S2. [file aac.01720-24-s0001.docx]

**Supplemental Data Section**

**Title: Siglec-Targeted Liposomes to Identify Sialoglycans Present on Fungal Pathogens**

Suresh Ambati, Quanita J. Choudhury, Jesse Ann Peter, Kelley W. Moremen, Digantkumar Gopaldas Chapla, Zachary A. Lewis, Xiaorong Lin, and Richard B. Meagher

**Supplemental Tables and Figures**

**Supplemental Table ST1. Example cognate ligands of SIG3 and SIG15.**

| **Siglec** | **Examples of Sialylated Ligands** | **Reference** |
| --- | --- | --- |
| SIG3/CD33 | N-linked glycans, α2-6 and α2-3 linked sialylated glycans, Neu5Acα2–3(6-O-sulfo)Galβ1–4GlcNAc (6′-Su-SLacNAc) and many artificially substituted variants | (1, 2) |
| SIG15 | O-linked & N-linked glycans, α2-6 and α2-3 linked sialylated glycans, (Sialyl-Tn structure Neu5Acα2-6GalNAcα and other structures containing a Neu5Acα2-6HexNAc determinant). | (2-4) |

**Supplemental Figure SF1. Polypeptide and DNA sequences**

**Siglec-3 human protein redesigned to tag DectiSomes.** Truncated protein derived from UniProt P20138.2. His6 tag is in bold. Flexible linker with lysine (K) residues for protein coupling to NHS reactive group of NHS-PEG-DSPE underlined. Plain font for Ig-like lectin carbohydrate recognition domain and stalk region of Siglec-3. Alanine (A) residue to help adjust sequence for subsequent DNA cloning prior to Stop codon. The signaling domain has been deleted. Siglec-3 a.a. residues 20 to 249. MW 28,961.4. pI 8.91 A280 1.17 to 1.18 (call it 1.175)

**HsSIG3 modified polypeptide employed**

MAGNFWLQVQESVTVQEGLCVLVPCTFFHPIPYYDKNSPVHGYWFREGAIISRDSPVATNKLDQEVQEETQGRFRLLGDPSRNNCSLSIVDARRRDNGSYFFRMERGSTKYSYKSPQLSVHVTDLTHRPKILIPGTLEPGHSKNLTCSVSWACEQGTPPIFSWLSAAPTSLGPRTTHSSVLIITPRPQDHGTNLTCQVKFAGAGVTTERTIQLNVTYVPQNPTTGIFPGDGSGKQETRAGSGSGSGKGKGSGSGMAHHHHHHYGA Stop

**HsSIG3 E.coli codon optimized DNA construct** subcloned into pET-45B for IPTG inducible expression in E. coli BL21 (GenScript. ATG and TAA (underlined) are the start and stop codons. Not shown are the 5’ and 3’cloning sites NcoI (CCATGG) with ATG as the start codon and PacI (TTAATTAA)respectively.

ATGGCTGGAAATTTTTGGCTACAAGTACAGGAGAGCGTGACCGTTCAAGAGGGTTTGTGCGTGCTGGTGCCGTGTACCTTCTTCCATCCGATTCCATATTACGACAAAAACAGCCCGGTACACGGCTACTGGTTTCGCGAGGGCGCGATCATCTCCCGCGACAGCCCGGTTGCTACCAACAAGCTGGATCAGGAAGTGCAAGAGGAGACGCAGGGCCGCTTTCGTTTACTGGGAGACCCGAGCCGCAACAACTGCAGCCTGTCCATTGTGGATGCGCGTCGTCGTGATAATGGCTCTTACTTCTTCCGCATGGAACGTGGTAGCACCAAGTACAGCTACAAATCCCCGCAGTTGAGCGTTCATGTTACCGACCTGACCCACCGTCCGAAGATTCTCATCCCGGGCACCCTGGAACCGGGTCATTCCAAAAACCTGACCTGCAGCGTCAGCTGGGCATGTGAACAAGGTACGCCACCGATCTTTTCTTGGCTGTCTGCGGCCCCGACCTCCCTTGGTCCGCGTACCACTCACAGCAGCGTGCTGATTATCACCCCGCGTCCTCAAGATCATGGCACCAATTTGACTTGCCAGGTTAAGTTTGCGGGCGCTGGCGTCACTACGGAGAGAACCATTCAGTTGAATGTTACCTATGTTCCGCAAAACCCGACCACGGGTATCTTCCCGGGTGACGGCAGCGGTAAACAGGAAACGCGTGCAGGTAGTGGCAGTGGGTCGGGTAAGGGCAAAGGCTCTGGTTCTGGTATGGCGCATCACCATCACCACCACTATGGTGCCTAA

**Siglec-15 human protein redesigned to tag DectiSomes.** Truncated human protein derived from Fasta File Accession & Version AAY40743.1 His6 tag is in bold. Flexible linker with lysine (K) residues for protein coupling to NHS reactive group of NHS-PEG-DSPE underlined. Plain font for Ig-like lectin carbohydrate recognition domain and stalk region of Siglec-15. Alanine (A) residue to help adjust sequence for subsequent DNA cloning prior to Stop codon. The signaling domain has been deleted. a.a. residues 28 to 263. ProtParam 28,248.6 MW. pI 8.84, A280 0.96 mg/mL

**MAG**TENLLNTEVHSSPAQRWSMQVPPEVSAEAGDAAVLPCTFTHPHRHYDGPLTAIWRAGEPYAGPQVFRCAAARGSELCQTALSLHGRFRLLGNPRRNDLSLRVERLALADDRRYFCRVEFAGDVHDRYESRHGVRLHVTAAPRIVNISVLPGPAHAFRALCTAEGEPPPALAWSGQALGNSLAAVRSPREGHGHLVTAELPALTHDGRYTCTAANSLGRSEASVYLFRFHGASGASTVA**GSGSGKGKGSGSG**HHHHHHYGT**A Stop**

**HsSIG15 E.coli codon optimized DNA construct** subcloned into pET-45B for IPTG inducible expression in E. coli BL21 (GenScript. Underlined ATG and TAA are the start and stop codons. Not shown are the 5’ and 3’cloning sites NcoI (CCATGG) with ATG as the start codon and PacI (TTAATTAA)respectively.

ATGGCTGGAACAGAAAACCTATTAAATACTGAGGTTCATAGCAGCCCGGCGCAACGTTGGTCCATGCAAGTTCCGCCGGAAGTGAGCGCAGAGGCTGGCGACGCTGCCGTTCTGCCGTGTACCTTTACCCATCCGCATCGTCACTACGACGGCCCTCTCACCGCGATCTGGCGTGCAGGCGAACCGTATGCAGGCCCGCAGGTTTTTCGCTGCGCAGCGGCGCGTGGTTCCGAGTTATGCCAGACCGCGCTTAGTTTGCACGGTCGCTTCCGCTTGCTGGGCAACCCGCGTCGTAATGATCTGAGCCTGCGTGTCGAACGTCTGGCTTTGGCGGATGATCGTCGCTACTTCTGCCGTGTGGAATTTGCAGGCGATGTGCACGACCGCTACGAATCTCGTCATGGTGTTCGTCTGCATGTAACGGCGGCTCCGCGCATCGTGAACATTAGCGTCCTGCCGGGTCCGGCCCATGCGTTCAGAGCTTTATGCACGGCGGAGGGTGAGCCGCCACCGGCGCTGGCTTGGTCTGGTCAGGCATTGGGCAACAGCCTGGCGGCTGTGCGCTCGCCGAGAGAAGGTCACGGCCACCTGGTTACCGCGGAGCTGCCAGCGTTGACCCACGACGGTCGTTACACCTGTACTGCGGCGAATAGCCTGGGCCGTAGCGAGGCCAGCGTTTATCTGTTTCGCTTCCACGGTGCAAGCGGTGCCTCGACCGTGGCCGGGTCCGGTTCCGGCAAAGGTAAGGGTTCTGGCTCCGGTCACCACCATCACCACCACTATGGTACAGCGTAA

**Supplemental Figure SF2. SDS PAGE analysis of crude *E. coli* extracts of IPTG induced protein and protein following affinity purification. A.** Coomassie stained 12% acrylamide gel analysis of the affinity purification of Siglec proteins. **B.** Histogram density scan of affinity purified Siglec-3 and Siglec-15 proteins modified for insertion into liposomes.

**~~
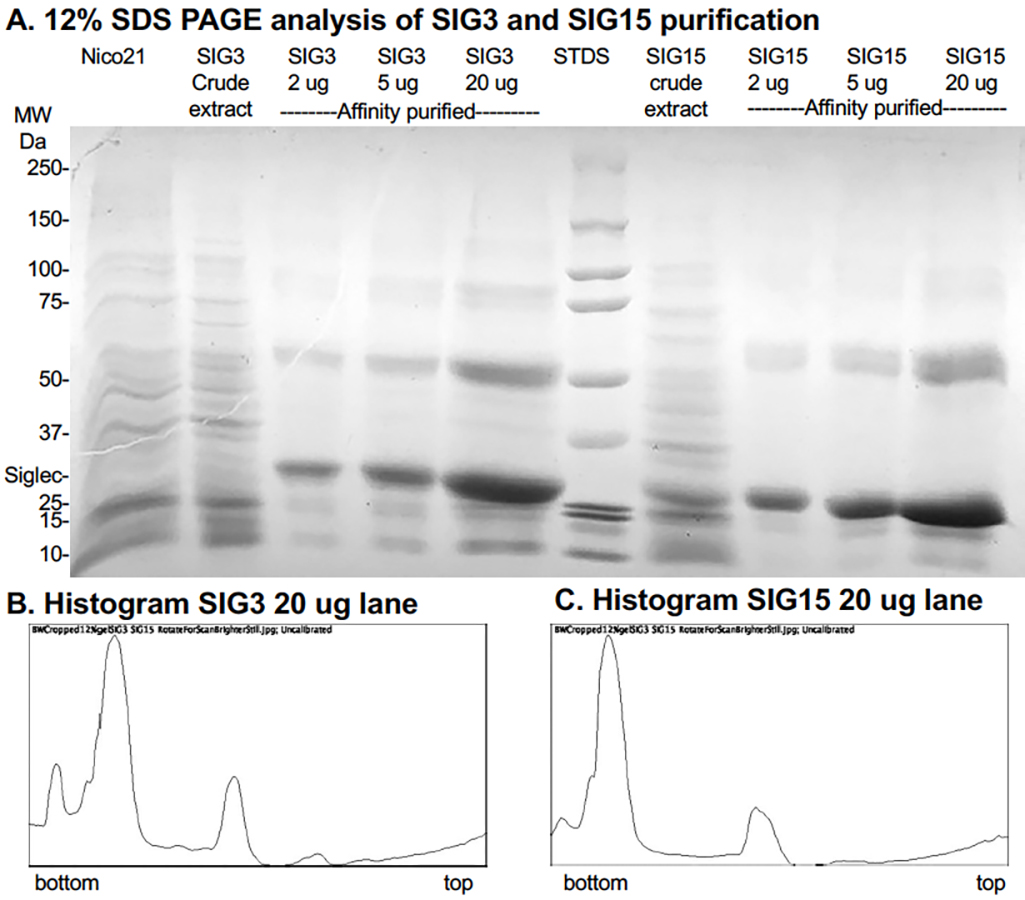
~~**

**References**

1. Rillahan CD, Macauley MS, Schwartz E, He Y, McBride R, Arlian BM, Rangarajan J, Fokin VV, Paulson JC. 2014. Disubstituted Sialic Acid Ligands Targeting Siglecs CD33 and CD22 Associated with Myeloid Leukaemias and B Cell Lymphomas. Chem Sci 5:2398-2406.

2. Bull C, Nason R, Sun L, Van Coillie J, Madriz Sorensen D, Moons SJ, Yang Z, Arbitman S, Fernandes SM, Furukawa S, McBride R, Nycholat CM, Adema GJ, Paulson JC, Schnaar RL, Boltje TJ, Clausen H, Narimatsu Y. 2021. Probing the binding specificities of human Siglecs by cell-based glycan arrays. Proc Natl Acad Sci U S A 118.

3. Anwar MT, Kawade SK, Huo YR, Adak AK, Sridharan D, Kuo YT, Fan CY, Wu HR, Lee YS, Angata T, Lin CC. 2022. Sugar nucleotide regeneration system for the synthesis of Bi- and triantennary N-glycans and exploring their activities against siglecs. Eur J Med Chem 232:114146.

4. Lenza MP, Egia-Mendikute L, Antoñana-Vildosola A, Soares CO, Coelho H, Corzana F, Bosch A, Manisha P, Quintana JI, Oyenarte I, Unione L, Moure MJ, Azkargorta M, Atxabal U, Sobczak K, Elortza F, Sutherland JD, Barrio R, Marcelo F, Jiménez-Barbero J, Palazon A, Ereño-Orbea J. 2023. Structural insights into Siglec-15 reveal glycosylation dependency for its interaction with T cells through integrin CD11b. Nature Communications 14:3496.
